# Rational Programming of Cas12a for Early-Stage Detection of COVID-19 by Lateral Flow Assay and Portable Real-Time Fluorescence Readout Facilities

**DOI:** 10.3390/bios12010011

**Published:** 2021-12-26

**Authors:** Zhijian Yi, Jean de Dieu Habimana, Omar Mukama, Zhiyuan Li, Nelson Odiwuor, Hanzhi Jing, Chengrong Nie, Mei Hu, Zuoxian Lin, Hongping Wei, Lingwen Zeng

**Affiliations:** 1 School of Food Science and Engineering, Foshan University, Foshan 528231, China; 2111958057@stu.fosu.edu.cn (Z.Y.); niecr@126.com (C.N.); 2 Key Laboratory of Regenerative Biology, South China Institute for Stem Cell Biology and Regenerative Medicine, Guangzhou Institutes of Biomedicine and Health, Chinese Academy of Sciences, Guangzhou 510530, China; jean@gibh.ac.cn (J.d.D.H.); omar@gibh.ac.cn (O.M.); li_zhiyuan@gibh.ac.cn (Z.L.); lin_zuoxian@gibh.ac.cn (Z.L.); 3 University of Chinese Academy of Sciences, 19 Yuquan Road, Shijingshan District, Beijing 100049, China; nelsonouma81@mails.ucas.ac.cn; 4 Department of Biology, College of Science and Technology, University of Rwanda, Avenue de l’armée, Kigali P.O. Box 3900, Rwanda; 5 CAS Key Laboratory of Special Pathogens and Biosafety, Centre for Biosafety Mega-Science, Wuhan Institute of Virology, Chinese Academy of Sciences, Wuhan 430071, China; 6 Sino-Africa Joint Research Centre, Nairobi 62000, Kenya; 7 College of Food Science and Technology, Huazhong Agricultural University, Wuhan 430070, China; jinghanzhi@126.com; 8 College of Food Science and Technology, Henan Agricultural University, 63 Nongye Road, Zhengzhou 450002, China; humei@henau.edu.cn; 9 Langyuan Biotechnology LLC, Foshan 528313, China

**Keywords:** COVID-19, clinical nasopharyngeal samples, CRISPR/Cas12a, RT-LAMP, portable real-time fluorescence detector, lateral flow strip

## Abstract

Coronavirus disease 2019 (COVID-19) caused by the SARS-CoV-2 virus has led to a global pandemic with a high spread rate and pathogenicity. Thus, with limited testing solutions, it is imperative to develop early-stage diagnostics for rapid and accurate detection of SARS-CoV-2 to contain the rapid transmission of the ongoing COVID-19 pandemic. In this regard, there remains little knowledge about the integration of the CRISPR collateral cleavage mechanism in the lateral flow assay and fluorophotometer. In the current study, we demonstrate a CRISPR/Cas12a-based collateral cleavage method for COVID-19 diagnosis using the Cas12a/crRNA complex for target recognition, reverse transcription loop-mediated isothermal amplification (RT-LAMP) for sensitivity enhancement, and a novel DNA capture probe-based lateral flow strip (LFS) or real-time fluorescence detector as the parallel system readout facility, termed CRICOLAP. Our novel approach uses a customized reporter that hybridizes an optimized complementary capture probe fixed at the test line for naked-eye result readout. The CRICOLAP system achieved ultra-sensitivity of 1 copy/µL in ~32 min by portable real-time fluorescence detection and ~60 min by LFS. Furthermore, CRICOLAP validation using 60 clinical nasopharyngeal samples previously verified with a commercial RT-PCR kit showed 97.5% and 100% sensitivity for S and N genes, respectively, and 100% specificity for both genes of SARS-CoV-2. CRICOLAP advances the CRISPR/Cas12a collateral cleavage result readout in the lateral flow assay and fluorophotometer, and it can be an alternative method for the decentralized field-deployable diagnosis of COVID-19 in remote and limited-resource locations.

## 1. Introduction

The novel coronavirus disease 2019 (COVID-19), caused by severe acute respiratory syndrome coronavirus 2 (SARS-CoV-2), poses a threat to global public health with a widespread infection of 236 million cases and 4.8 million deaths as of 5 October 2021 [1]. The SARS-CoV-2 outbreak has an evolutionary genetic connection with the coronaviruses that caused SARS-CoV and MERS-CoV pneumonia in Guangdong Province in 2003 and in the Middle East in 2012, respectively [2]. 

Currently, there is no proven treatment for COVID-19; however, several vaccines have been approved for emergency use, and others are still under clinical trials [3,4]. Given that vaccinated individuals remains infectious while asymptomatic, it is still vital to develop highly sensitive and user-friendly detection tools to counter pandemic transmission [5,6]. Although several diagnostic techniques, including molecular techniques (polymerase chain reaction, isothermal amplification, and CRISPR/Cas-based techniques) and serological tests, have been developed for SARS-CoV-2 detection, several of them have not yet been approved for clinical use. As such, various parts of the globe are still relying on laboratory-centered instruments—PCR, clinical symptom assessment, and body temperature monitoring, which are far beyond meeting the requirements of epidemiological surveillance [7,8]. 

Reverse transcription-polymerase chain reaction (RT-PCR) is widely used as a clinically approved and gold standard assay to diagnose SARS-CoV-2. However, RT-PCR requires a long turnaround time, skilled operating staff, huge thermocyclers, and a well-established laboratory, confining its point-of-care (POC) application [9]. Isothermal amplification techniques have been acquired to circumvent the limitations of PCR [10]. Particularly, reverse transcription loop-mediated isothermal amplification (RT-LAMP) has been used for SARS-CoV-2 diagnosis [8]. More importantly, RT-LAMP only requires a constant temperature for the entire process and is reported to be 100 times more sensitive than RT-PCR. However, existing RT-LAMP strategies for SARS-CoV-2 detection typically rely on pH indicators or dyes that are susceptible to false positives. A turbidimeter is also required to interpret the findings, increasing the application costs and limiting their use for POC [7]. Moreover, although serological tests are used to diagnose active infection, they count on the detection of immunoglobulin M (IgM) and immunoglobulin G (IgG) antibodies, which are post-infection markers, while antigen testing could face low sensitivity, impacting the overall traceability of patients [10].

CRISPR/Cas systems defend bacteria and archaea against invading phages, and this propensity has been harnessed in gene editing and molecular diagnostics [11]. CRISPR/Cas systems are classified into two broad classes (class1 and class 2), which are further categorized into six subtypes, with several orthologs found across the microbial community’s diverse range of organisms [12]. Unlike class 1 CRISPR/Cas systems (types I, III, and IV) which employ a multi-protein cascade to acquire interference, class 2 CRISPR/Cas systems (types II, V, and VI) utilize a single multi-domain protein, such as Cas9, Cas12, Cas13, and Cas14, and are the most frequently used in bioengineering and nucleic acid detection [13,14]. Generally, a CRISPR/Cas system involves two main parts: the guide RNA complementary to the target sequence, which recognizes it, and a Cas enzyme, which binds and cuts the target. In contrast to the Cas9 gene editor, Cas12, Cas13, and Cas14 exhibit a collateral cleavage activity, a propensity used in diagnostics [15,16]. CRISPR/Cas12 system uses a complex of guide-RNA-bound Cas12 nuclease (Cas12a/crRNA) that becomes activated upon binding the target of interest and enables the collateral cleavage of any single-stranded DNA (ssDNA) in the vicinity [17,18]. This ssDNA oligonucleotide is modified with biological tags and solely becomes detectable via fluorescence, colorimetric, or electrochemical signaling upon *trans*-cleavage [19,20]. For example, the groups of Doudna [21] and Zhang [22] utilized Cas12 and Cas13 to develop DNA endonuclease-targeted CRISPR *trans* reporter (DETECTR) and specific high-sensitivity enzymatic reporter unlocking (SHERLOCK), respectively. DETECTR and SHERLOCK assays are highly sensitive, specific, and cost-effective, and they generate the results in a shorter time than that required in PCR. These assays have also received Emergency Use Authorisation (EUA) for SARS-CoV-2 detection; however, they should only be performed by authorized clinical laboratory under the Food and Drug Administration’s EUA [23,24].

Due to its convenience with CRISPR/Cas systems and isothermal amplification techniques, a paper-based diagnostic tool known as the lateral flow strip (LFS) serves as a promising tool that can satisfy the criteria for early-stage diagnosis of any unpredicted pandemic outbreak [25,26]. An LFS is commercially cheap, portable, and user-friendly, and it allows the high-throughput detection of massive samples in parallel. For instance, several researchers, including Broughton et al. [27] with the DETECTR assay, Ali et al. [28] with the iSCAN assay, Nguyen et al. [29] with the ENHANCE assay, and Ooi et al. [30] with the VaNGuard assay, used Milenia HybriDetect dipsticks for SARS-CoV-2 detection. The HybriDetect dipstick works on the principle of switching a test line with a control line for result interpretation. This switching mechanism of the HybriDetect dipstick has disadvantages in that it can lead to intuitive misinterpretation due to the competitive binding of the reporter to the control and test lines. More recently, Gong et al. [31] demonstrated a novel reporter in a method named TESTOR (Targeting DNA by Cas12a-based Eye Sight Testing in a Onepot Reaction) to solve the unspecificity of the HybriDetect dipstick. The concept of TESTOR is similar to that of the HybriDetect dipstick; however, the difference lies in TESTOR’s novel reporter, which was designed with a phosphorothioate in the middle of the digoxin and biotin labels. Zhu et al. [25] also established a CRISPR/Cas12a-based detection system using multiple cross displacement amplification (MCDA) and an LFS for COVID-19 detection. Similar to the HybriDetect dipstick, an FITC-biotinylated reporter was employed; however, the conjugate pad, test, and control lines were modified with streptavidin–gold, biotin–bovine serum albumin, and anti-FITC, respectively. Despite the advancements of the CRISPR/Cas12a-based lateral flow assay (LFA), these assays could lead to nonspecific readouts due to the switching mechanism of the test and control lines. 

Herein, we report a novel alternative assay for SARS-CoV-2 diagnosis to advance the CRISPR/Cas12a-based LFA. We demonstrate a cost-effective CRISPR/Cas12a approach for COVID-19 detection using RT-LAMP to boost the sensitivity and LFS or real-time fluorescence as an independent parallel system for result readout, termed CRICOLAP. By targeting the surface glycoprotein (S)- and nucleocapsid phosphoprotein (N)-encoding genes of SARS-CoV-2, we design a biotinylated reporter and optimized its complementary capture probe in an LFA. In the presence of the target SARS-CoV-2, the reporter is *trans*-cleaved and fails to hybridize with the capture probe, indicating a positive readout. Our novel LFS shows two bands for negative samples and one band for positive samples. The results are also confirmed using a real-time portable Dhelix-Q5 isothermal fluorescence PCR machine (hereafter referred as Dhelix-Q5 for simplicity), but using a fluorescently labeled reporter. Thus, the developed approaches could be used for the rapid, accurate, and inexpensive detection of infectious diseases.

## 2. Materials and Methods

### 2.1. Materials and Reagents

All oligonucleotide sequences (Supplementary Materials, Tables S1–S4) were synthesized by Sangon Biotech (Shanghai, China). Bst 2.0 DNA polymerase, Avian Myeloblastosis (AMV) reverse transcriptase, high purity LbaCas12a, NEB buffer 2.1, and deoxynucleoside triphosphates (dNTPs) were obtained from New England Biolabs (Ipswich, MA, USA). Streptavidin (SA), tetrachloroauric(III) acid (HAuCl_4_aq), and trisodium citrate were purchased from Sigma–Aldrich (Steinheim, Germany). Fiberglass, nitrocellulose membrane, and absorbent papers were from Millipore (Billerica, MA, USA). The dispenser machine was purchased from Shanghai Kinbio (Shanghai, China). The portable Dhelix-Q5 isothermal fluorescence PCR machine (portable Dhelix-Q5) was purchased from Guangzhou international biological island (Guangzhou, China). The remaining reagents, such as buffers, were prepared in our laboratory. 

### 2.2. crRNA Design, Primer Selection, and RT-LAMP Assay

Both target genes were cloned into the pcDNA3.1+ vector. The N gene was cloned into the pcDNA3.1+ vector at the HindIII/EcoRI site, while the S gene was inserted behind the N gene at the EcoRI/XhoI site to obtain a fused S + N sequence (Table S1). The crRNAs targeting the S and N genes (Table S2) were designed using Benchling online software, and the ones with a higher score were selected. Premier Biosoft and PrimerExplorer V5 online software were used to design LAMP primers (Table S3) and specifically confirmed using the NCBI-BLAST database. RT-LAMP reaction was performed following the NEB protocol (New England, USA). Typically, 25 µL of each reaction contained 10× isothermal amplification buffer; 6 mM of MgSO4; 8 U/µL of Bst 2.0 DNA polymerase; 7 U/µL of AMV reverse transcriptase; primers F3/B3 (0.2 µM), FIB/FIP (1.6 µM), and Loop F/Loop B (0.4 µM); 1.4 mM dNTPs; 2 µL of RNA target; and water to the final volume. The mixture was sealed and incubated at 65 °C for 25 min in a water bath. For fluorescence detection, SYTO-9 was added, and this was followed by incubation in a Dhelix-Q5 (λ_ex =_ 470 nm; λ_em =_ 520 nm).

### 2.3. Target Cleavage, Lateral Flow, and Fluorescence Detection Assays

The CRISPR/Cas12a reaction for target and reporter cleavage was performed following a previous method with minor modifications [21]. Within a 20 µL mix, the reaction included 10 µL of 2× cleavage buffer [1% glycerol, 10 mM MgCl_2_, 150 mM KCl, 0.5 mM DTT, 20 nM HEPES (pH 7.5)], 50 nM of Cas12a, 500 nM of fluorophore–quencher ssDNA-labeled reporter (FQ-ssDNA reporter), 40 nM of activator, 50 nM of crRNA, 1 µL of target amplicons, and sterile nuclease-free water. After mixing thoroughly, the mixture was incubated at 37 °C for 30 min to ensure sufficient cleavage. The *trans*-cleavage for fluorescence detection was monitored using a Dhelix-Q5. For the LFA, the protocol was the same; however, the FQ-ssDNA reporter was replaced by our customized biotinylated ssDNA reporter (bio-ssDNA reporter) (Table S4), and the cleavage activity on the LFS was monitored by the naked eye.

### 2.4. Preparation of Gold Nanoparticles, Lateral Flow Strip Design, and Assembly

A complete LFS basically consists of five main parts: the sample pad, conjugate pad, nitrocellulose membrane (NC), absorbent pad, and backing pad [19]. The sample pad fabricated from glass fibers was thoroughly wetted with a buffer of pH 8.0 (2% glucose, 1% Triton, 50 mM boric acid, 1% BSA), dried, and then kept in a desiccant container. The conjugate pad, also made of glass fibers, was embedded with the AuNP-SA solution [32] and dried thoroughly before use. Using a dispenser machine, the test and control lines on the NC membrane were sprayed with a customized DNA capture probe and biotinylated rabbit polyclonal anti-IgG antibody solutions, respectively. The distance between the test and control lines was estimated to be 7 mm, and then the NC membrane (25 mm in width) was dried and kept at room temperature for 12 h and 4 °C until further use. Lastly, all the LFS parts were collectively attached, each overlapping by 2 mm. Finally, the LFS with a 4 mm width was readily obtained using an LFS cutter.

### 2.5. Clinical Sample Preparation, RNA Extraction, and Ethics Statement

Nasopharyngeal swabs stored in viral transport medium (VTM) at the Wuhan Institute of Virology (WIV), Chinese Academy of Sciences, were inactivated in a biosafety level 2 laboratory by heating at 95 °C for 5 min. The total RNA was extracted from the inactivated samples using a magnetic bead-based extraction kit, FineMag Quick Viral DNA/RNA (Genfine Biotech, Changzhou, China), on an automated device, Purifier Modesty (Genfine). China CDC authorized WIV to handle COVID-19 patients’ samples during the outbreak in Wuhan, China.

### 2.6. RT-qPCR Amplification 

The RT-qPCR was performed using a commercial kit from Sansure Biotech (Changsha, China) officially approved for COVID-19 testing. The kit targets ORF1ab and the N gene of SARS-CoV-2 together with the human internal control gene (IC). RT-qPCR was conducted in a 50 µL reaction tube consisting of 26 µL of reaction solution, 4 µL of enzyme mixture, and 20 µL of template RNA. The reaction was performed on a CFX96 DeepWell real-time system (BIORAD, Hercules, CA, USA). The thermocycling protocol was set as follows: reverse transcription was proceeded at 50 °C for 30 min, and cDNA pre-denaturation occurred at 95 °C for 1 min, followed by 45 cycles at 95 °C for 15 s. The annealing and fluorescence acquisition steps were conducted at 60 °C for 30 s. A sample was considered positive with a cycle threshold (Ct) of less than 40, and the test was valid only if the cycle threshold (Ct) was less than 35 (Ct < 35).

## 3. Results

### 3.1. Principle of CRICOLAP Real-Time Fluorescence and LFS Detection of COVID-19

**Scheme 1:**
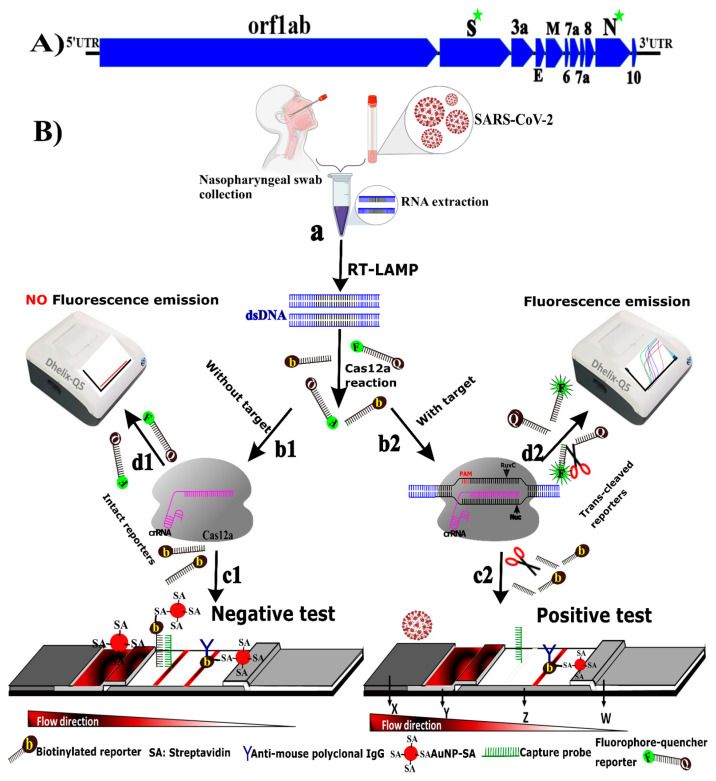
(**A**) SARS-CoV-2 genome mapping showing the localization of different genes (target genes are marked with a green star). (**B**) Illustration of the CRICOLAP for visual and fluorescence detection of SARS-CoV-2. T: test line/band; C: control line/band; X: sample pad; Y: conjugate pad; Z: nitrocellulose membrane; W: absorbent pad. This figure was created with BioRender.Com and INKSCAPE software.

In the present study, CRICOLAP was established by integrating the CRISPR/Cas12a coupled with RT-LAMP to boost the sensitivity and two independent parallel systems (LFS and real-time fluorescence) for result readout. This study targeted the S and N conserved genes of SARS-CoV-2 (Scheme 1A).

As shown in Scheme 1B, the extracted RNAs were RT-LAMP pre-amplified (Scheme 1B, a) and applied to Cas12a reaction for collateral cleavage (Scheme 1B, b1 and b2) and then to the LFS or real-time fluorescence reader for results readout. For the LFS, we customized a novel LFA based on the CRISPR/Cas12a *trans*-cleavage mechanism. Researchers have shown that a DNA probe of more than 15 bases generates a strong signal on the LFS via DNA hybridization [33]. Furthermore, the CRISPR/Cas12-based FQ-ssDNA reporter (FQ-TTTATT/FQ-3TA2T) was reported to release fluorescence upon *trans*-cleavage by Cas12a [21]. Therefore, we sought to customize the fluorescent reporter for use in our LFS. From various attempts (Table S4), we realized that a biotinylated-ssDNA reporter of 11 bases (Bio-TTTTTTTTATT/bio-8TA2T) could strongly hybridize with a 32-base ssDNA probe sequence (Table S5) on the LFS test line; however, it was undetectable once cleaved by Cas12a (Figure S1). Therefore, when the CRISPR/Cas12a mixture is subjected to the sample pad, it migrates to the conjugate pad containing the AuNP-SA complex and flows toward the test line and control line across the NC membrane. The aggregation of the bio-ssDNA reporter at the test line reveals a negative test (Scheme 1B, c1), while the disappearance of a visual red-like test line indicates a positive test resulting from efficient reporter *trans*-cleavage (Scheme 1B, c2). In parallel, for fluorescence analysis, the FQ-ssDNA reporter remained intact due to the absence of the target (Scheme 1B, d1), while in the presence of the target, the FQ-ssDNA reporter fluoresced gradually as a result of *trans*-cleavage (Scheme 1B, d2).

### 3.2. Method Performance Optimization

**Figure 1:**
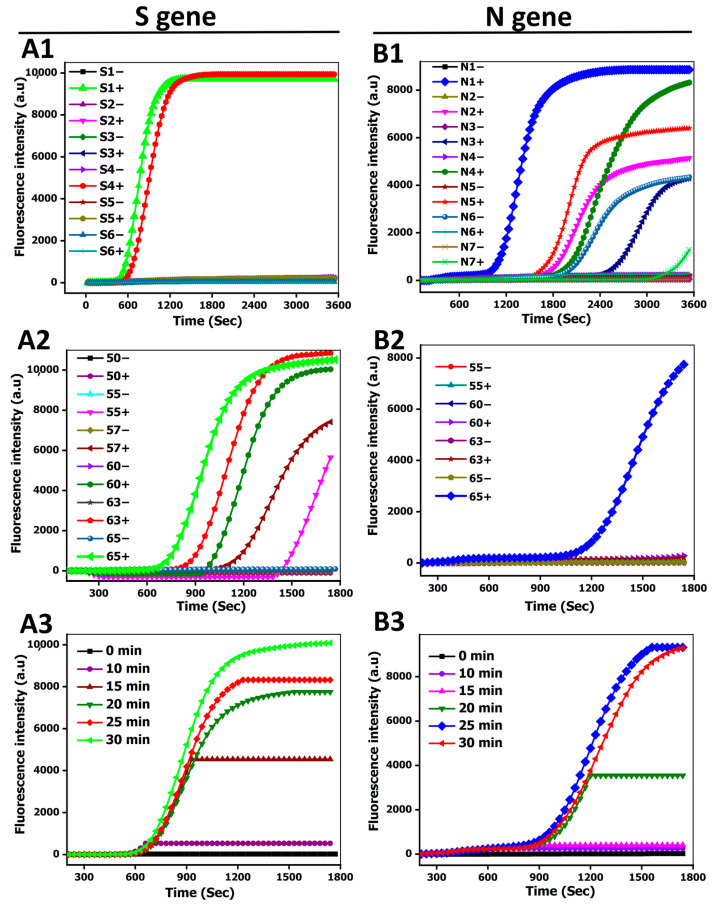
CRICOLAPʹs RT-LAMP optimization. Real-time fluorescence analysis of RT-LAMP products for S and N genes. The signs “+” and “−” denote the reaction with the target and no-template, respectively. S gene (**A1**) and N gene (**B1**) primer selection. Temperature optimization (**A2**,**B2**). Reaction time optimization (**A3**,**B3**).

The RT-LAMP and CRISPR/Cas12a experimental conditions were optimized to improve the *trans*-cleavage efficiency. Firstly, six and seven different primer sets (Table S3) for the S and N genes were used in RT-LAMP. Of the six sets of the S gene, the S1 and S4 primer sets demonstrated excellent amplification in electrophoresis (Figure S2a) and real-time fluorescence assays (Figure 1A1). However, S1 showed a fast response and was selected for further experiments. For the N gene, the N1 set was selected (Figure S2b and Figure 1B1). For the temperature assessment, RT-LAMP was tested from 55 to 65 °C, and a maximum signal was obtained at 65 °C (Figure S2c,d; Figure 1A2,B2). The amplicons could be observed within the first 10 to 25 min (Figure S2e,f; Figure 1A3,B3).

**Figure 2:**
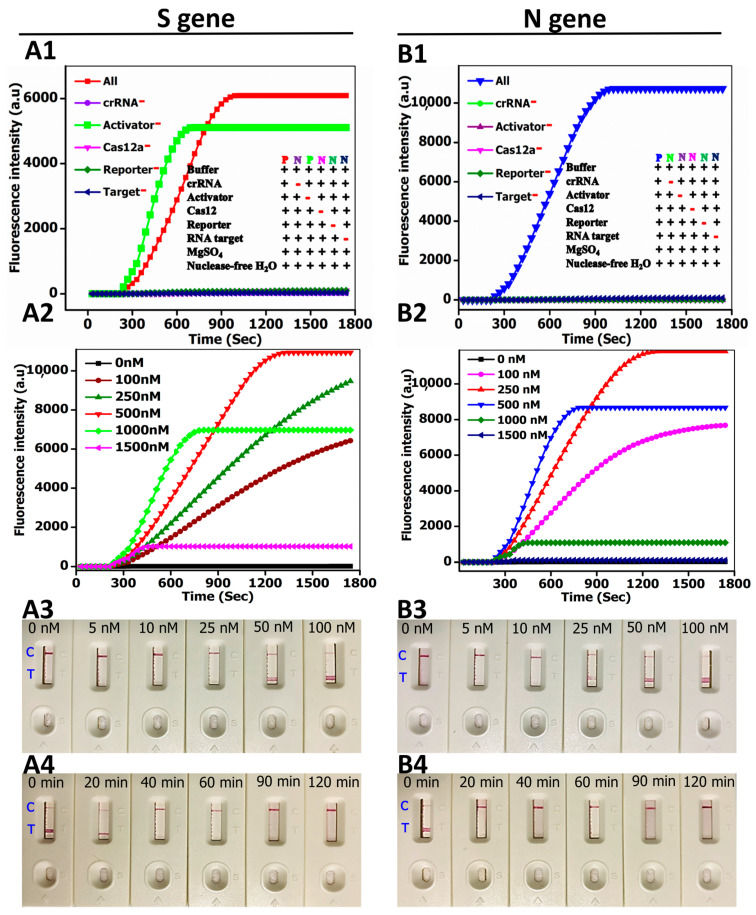
CRICOLAPʹs CRISPR reaction optimization. Evaluation of CRISPR premix for S (**A1**) and N genes (**B1**). The signs “+” and “−” denote the presence or absence of one component in the reaction, respectively. Fluorescence intensity corresponding to different concentrations of FQ-ssDNA reporter (**A2**,**B2**). LFS signals corresponding to different concentrations of bio-ssDNA reporter (**A3**,**B3**) and CRISPR response time (**A4**,**B4**).

Three crRNAs for each target gene were designed, and the ones with a higher score and target matching efficiency were selected (Figure S3a,b). For the CRICOLAP validation, we performed separate reactions by omitting individual components in the CRISPR reaction (crRNA, activator, Cas12a enzyme, reporter, and the target). Our assay was validated by confirming the ability of crRNA to guide Cas12a to *trans*-cleave the reporter in the presence of all reaction components. There was no reporter *trans*-cleavage in the absence of one reaction component except for the S gene activator (Figure 2A1), presuming that the S gene activated Cas12a per se [21,34]. Moreover, the cleavage could not occur in the absence of the activator for the N gene (Figure 2B1). The optimum reporter concentrations were 1000 nM and 500 nM for the S and N genes, respectively (Figure 2A2,B2).

Subsequently, the reporter concentrations varying from 0 nM to 100 nM were assessed for LFA. An intense test line was observed at 25 nM and 10 nM for S and N genes, respectively, in the absence of the target and no test line in the presence of the target (Figure 2A3,B3). Finally, we assessed the required time for a complete *trans*-cleavage that can result in a non-detectable test line upon hybridization of the reporter to the capture probe. Forty and twenty minutes were enough to ensure the complete cleavage of a bio-ssDNA reporter (Figure 2A4,B4). However, a detectable signal at the LFS resulting from hybridization could be observed within 5 min.

### 3.3. CRICOLAP Sensitivity

**Figure 3:**
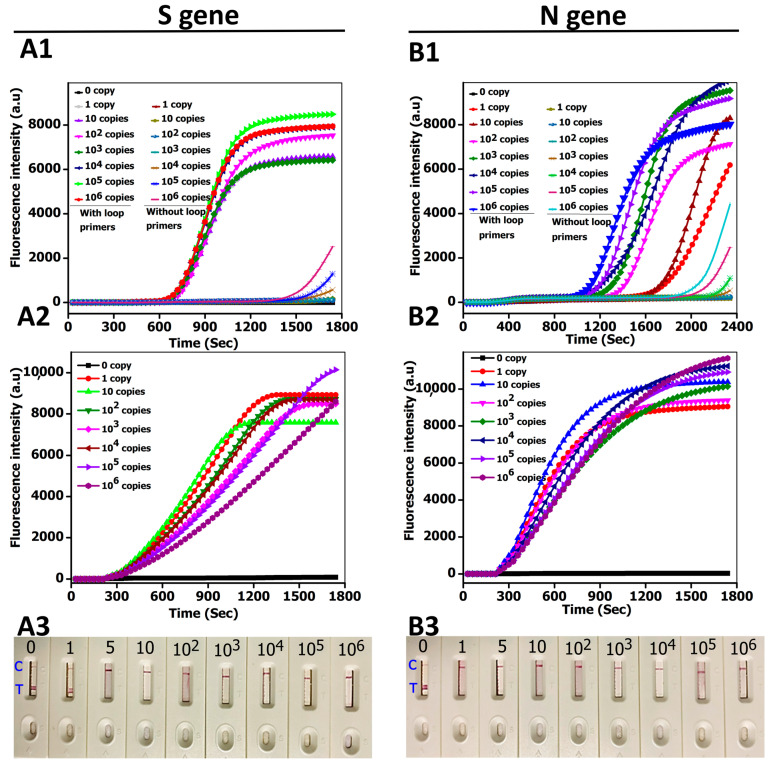
CRICOLAP sensitivity evaluation. RT-LAMP sensitivity with or without loop primers for S (**A1**) and N genes (**B1**). CRICOLAP sensitivity monitoring via real-time fluorescence analysis (**A2**,**B2**) and LFA (**A3**,**B3**). For RT-LAMP and CRISPR cleavage assays, each reaction was performed using 2 µL and 1 µL, respectively, using the following serial dilutions: 0, 1, 10, 10^2^, 10^3^, 10^4^, 10^5^, and 10^6^ copies/µL.

We assessed the efficiency of four and six LAMP primers. The four primers alone without loop primers achieved a limited sensitivity, while the sensitivity drastically increased with loop primers (Figure 3A1,B1; Figure S4a,b). This activity could be attributed to the LAMP’s rolling cycle amplification [35]. Next, the overall CRICOLAP sensitivity was further evaluated using real-time fluorescence analysis and LFA. First, various concentrations of RT-LAMP amplicons ranging from 0 to 10^6^ copies/µL were investigated. Our method achieved one copy sensitivity for both the S and N genes (Figure 3A2,B2). Second, by replacing FQ-ssDNA with a bio-ssDNA reporter, similar sensitivity was achieved for LFA except for the S gene, which could not attain one copy resolution. CRICOLAP’s LFS identified as low as five copies/µL (Figure 3A3) and one copy/µL (Figure 3B3) for the S and N genes, respectively. Intriguingly, there was no significant difference in sensitivity between the synthetic RNA and the RNA extracted from patients’ samples, which is in accordance with Ding et al. [36].

CRICOLAP sensitivity aligns with the recent CRISPR/Cas12-isothermal amplification-based techniques, but with different concepts [37,38]. For instance, two different groups [27,28] reported an LOD of 10 copies/µL using CRISPR/Cas12a, LAMP, and commercial Milenia HybriDetect dipsticks for colorimetric detection and a laboratory-centered fluorophotometer. Similarly, by substituting LAMP with RPA, Talwar et al. [2] and Gong et al. [31] also achieved LODs of 10 and 5 copies/reaction, respectively. In the HybriDetect dipstick, a test line is immobilized with an anti-goat antibody that captures AuNPs with goat anti-FITC antibody, whereas the control line is coated with a streptavidin that captures the FITC-biotinylated reporter. Upon *trans*-cleavage, a biotin-labelled reporter fails to capture AuNPs-goat anti-FITC antibody, thereby forming a test line. This switching format has disadvantages in that it can lead to intuitive misinterpretation due to the competitive binding of the reporter to the control and test lines, a limitation that CRICOLAP overcomes.

### 3.4. CRICOLAP Specificity

**Figure 4:**
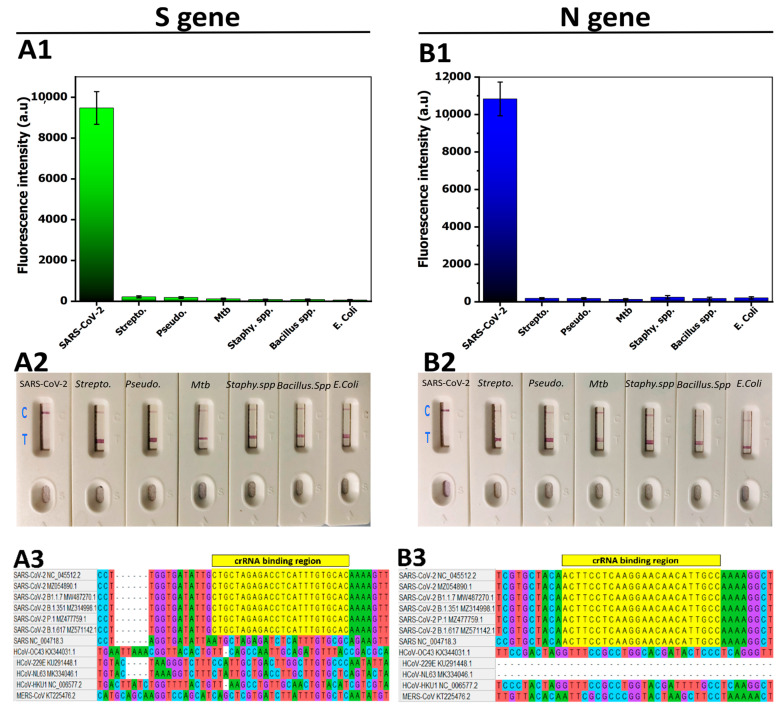
Specificity assessment of CRICOLAP. Validation of fluorescence assay (**A1**,**B1**) and LFA (**A2**,**B2**). The positions of S (**A3**) and N (**B3**) crRNAs (ScrRNA1 and NcrRNA2) from six SARS-CoV-2 strains including two wildtypes (SARS-CoV-2 NC_045512.2 and SARS-CoV-2 MZ054890.1), four common variants (SARS-CoV-2 B1.1.7 MW487270.1, SARS-CoV-2 B.1.351 MZ314998.1, SARS-CoV-2 P.1 MZ477759.1, and SARS-CoV-2 B.1.617 MZ571142.1), and six additional human coronaviruses (SARS NC_004718.3, HCoV-OC43 KX344031.1, HCoV-229E KU291448.1, HCoV-NL63 MK334046.1, HCoV-HKU1 NC_006577.2, and MERS-CoV KT225476.2). MEGA software was used to align sequences of several coronaviruses acquired from GenBank. Positions that are perfectly aligned to crRNAs are highlighted in yellow.

To assess the specificity of CRICOLAP, various species (*Streptococcus pneumoniae, Pseudomonas aeruginosa, Mycobacterium tuberculosis, Staphylococcus aureus, Bacillus* spp., and *Escherichia coli*) that usually infect the human respiratory tract and impair the breathing system in a similar manner to SARS-CoV-2 were examined. As shown in Figure 4A1,A2,B1,B2, the cleavage occurred only in the SARS-CoV-2 target containing samples, proving no cross-reactivity with other species. We also used MEGA X software to align the crRNAs (ScrRNA1 and NcrRNA2) with SARS-CoV-2 variants and other human coronavirus genomes (Figure 4A3,B3). Interestingly, we found that our crRNAs completely matched with all the SARS-CoV-2 variants (SARS-CoV-2 B1.1.7 alpha, SARS-CoV-2 B.1.351 beta, SARS-CoV-2 P.1 gamma, and SARS-CoV-2 B.1.617 delta), indicating a strong specificity of our crRNAs. Moreover, we noticed that they were not related to other viral sequences. Unfortunately, NcrRNA2 appeared to perfectly align with SARS NC_004718.3 (Figure 4B3); however, NcrRNA3 could not (result not shown), which may suggest the avoidance of false positives. Furthermore, because crRNAs have been demonstrated to be sensitive to single-base mismatch, the crRNAs targeting the S and N genes of SARS-CoV-2 had many mismatches (more than three mismatches) to other coronaviruses, indicating the excellent specificity of CRICOLAP.

### 3.5. CRICOLAP Validation in Clinical Samples

**Figure 5:**
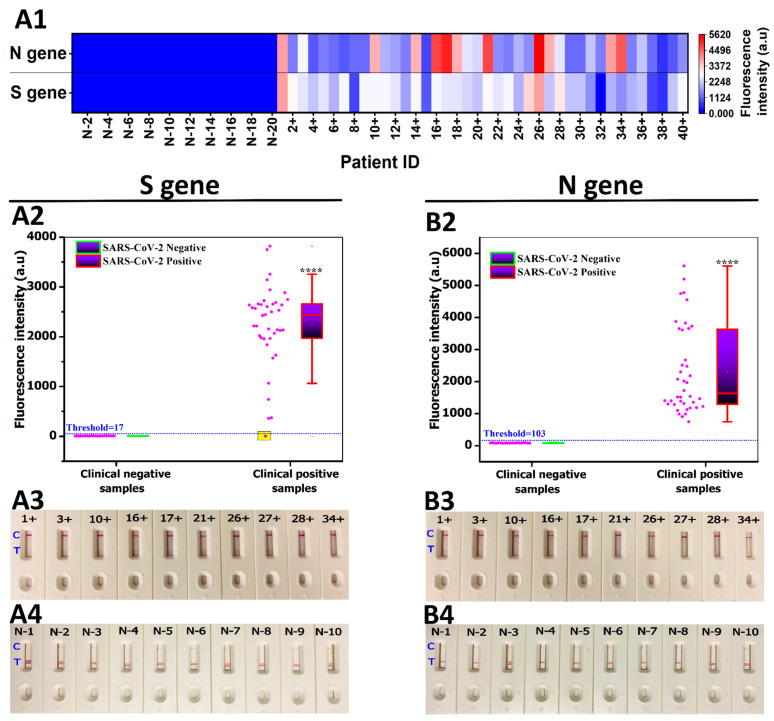
Validation of CRICOLAP for SARS-CoV-2 detection in 60 nasopharyngeal samples. Validation of fluorescence assay, results indicated using a heat map (**A1**) with corresponding box plot data distribution (**A2**,**B2**) based on the three standard deviations above the mean BGF at 7 min of the validated negative controls, *n* = 20, **** *p* < 0.0001; two-tailed Student’s *t*-test. For LFA, only 10 samples are shown for each gene; S gene (**A3**,**A4**), N gene (**B3**,**B4**), the remaining samples are found in the supporting information, Figures S6 and S7. Samples denoted with “+” and “N-” are from positive and negative clinical samples, respectively.

To validate the feasibility of CRICOLAP, 60 clinical patients’ samples were examined (namely, 40 positive and 20 negative samples) initially verified by a commercial RT-PCR kit (Figure S5). As shown by a heat map, Figure 5A1, the fluorescence intensity drastically increased within all the positive clinical samples due to the FQ-ssDNA reporter *trans*-cleavage, implying the presence of SARS-CoV-2 (Figure S5). The distribution of corresponding data is indicated by the box and whisker plots (Figure 5A2,B2) and is depicted based on the detection threshold generating fluorescence intensity in a timely manner dependent on the CRISPR reaction (7 min, *n* = 6). The significant threshold fluorescence value (denoted with a horizontal dashed line, Figure 5A2,B2) calculated based on the three standard deviations above the mean background fluorescence (BGF) [39] was obtained at 7 min of the validated negative controls (*n* = 20), the highest recorded BGF being 12.3 and 96 for S and N genes, respectively. Therefore, the sample was confirmed positive if it was above 17 for the S gene and above 103 for the N gene, and negative otherwise. Of the 40 positive samples examined, patient sample numbers 38 and 39 were negative for the ORF1ab gene tested by a commercial RT-PCR kit (Figure S5a); however, they were identified to be positive for N and IC genes as well as S and N genes by the use of CRICOLAP.

In parallel, the LFS showed no visual test lines for the ten positive clinical samples (Figure 5A3,B3). However, a visible test line was detected by the naked eye in the ten negative samples due to the failure of *trans*-cleavage, which resulted in the hybridization of the reporter and capture probe at the test line (Figure 5A4,B4). CRICOLAP showed 97.5% and 100% sensitivity for S and N genes and 100% specificity consistency with the validated commercial RT-PCR data for both negative and positive patient samples (Table S6).

## 4. Discussion

In the wake of the raging COVID-19 pandemic, an increasing demand for highly effective diagnostics for SARS-CoV-2 detection has been observed. With the breakthrough utilization of CRISPR/Cas in diagnosis based on the *trans*-cleavage of the ssDNA reporter in the presence of a target [19], we successfully customized a novel CRISPR reporter for use in LFA and fluorophotometry in the assay termed CRICOLAP. The assay accurately identified SARS-CoV-2 from 60 clinical patients’ samples initially confirmed positive or negative by a commercial RT-PCR kit approved by China CDC. Our novel LFS shows one band and two bands for SARS-CoV-2-positive and -negative patients’ samples, respectively, unlike the conventional LFA. 

Coupling any detection assay with an on-site visual readout is essential, particularly when equipment is not affordable. CRICOLAP LFS is cheap (USD 0.1, excluding the CRISPR reagents) (Table 1) and can be deployed for on-site testing. According to Milenia [40] and Gang et al. [31], the detection mechanism of switching the test and control lines in Milenia HybriDetect may sometimes lead to nonspecific signal readouts at the test line, because both test and control line signals depend on the reporter *trans*-cleavage. This may make discerning the bands of weak positive patient samples from the bands caused by nonspecific faint bands challenging. To solve this, we successfully customized a reporter that complements an optimized capture probe. As such, the signal at the test line could solely be obtained without switching the test and control lines to prevent intuitive misinterpretation. More importantly, since CRISPR/Cas12a machinery relies on three main elements (Cas12a, crRNA, and the reporter) to achieve nucleic acid detection, we were able to design and transduce the signal directly from the CRISPR/Cas12a machinery without using an auxiliary carrier, such as biotin, streptavidin, or digoxin, as observed in several CRISPR/Cas12a-based LFA [2,25,27,28,29,30,31] (Table 1). The concept of CRICOLAP LFS can also serve as a state of the art to harness the trans-cleavage activity, which we believe can increase sensitivity and specificity, because the signal reporter of the CRISPR/Cas machinery is directly captured by a signal amplifier (capture probe) at the test line. On the other hand, the Dhelix-Q5 Isothermal Fluorescence PCR instrument is portable (~4.3 kg) and ten to fifteen times cheaper than RT-qPCR, and it can monitor real-time fluorescence detection with a high signal-to-background. Moreover, it does not require thermocycling protocols as it uses a constant temperature to generate the results. Both methods of CRICOLAP hold potential due to their abilities to detect few copies of S or N genes of SARS-CoV-2 in clinical nasopharyngeal swabs.

CRICOLAP LFS may be hampered by false positives from spurious amplicons and unsuccessful reporter *trans*-cleavage. These can be prevented by appropriate reagent manipulation and proper incubation time monitoring, respectively. Additionally, the LAMP cross-contamination can be eliminated through a two-step loading of primers, with forward, backward, and loop primers within the first 10 min, followed by inner primers. This system stability in SARS-CoV-2 detection promises one-pot feasibility toward minimizing the risks of spurious contamination and repeated reagent manipulation. Another attractive feature to achieve one-pot detection would be the integration of a thermostable enzyme such as *Alicyclobacillus acidophilus* Cas12b (AapCas12b) and LAMP since AapCas12b retains optimum nuclease activity over a wide range of temperatures (31–59 °C) [41]. This can also facilitate automation, using a custom, handheld cartridge to streamline the testing workflow, thereby enabling POC testing in remote settings. In the future, CRICOLAP could emerge as a robust alternative assay to provide timely support in the battle against infectious diseases in remote and limited-resource areas.

## Figures and Tables

**Table 1:** Comparison of currently reported CRISPR/Cas12 lateral flow assays with the CRICOLAP assay for SARS-CoV-2 detection.

Assay	Target Gene	Amplification Method	Amplification Time	Total Assay Time	Detection Limit	Assay Control	Control Line	Test Line	LFS Source	LFS Price	Ref.
CRICOLAP	N and S genes	LAMP	25 min	60 min	1 copy/μL	LFB, fluorescence	Biotinylated antibody	ssDNA probe	Designed and assembled by our group	USD 0.095	This study
DETECTR	E and NP genes	LAMP	30–40 min	60 min	10 copies/μL	LFB, fluorescence	Streptavidin	Anti-conjugate antibody	Purchased from Milenia Biotech.	USD < 3	[27]
VaNGuard	ORF1b, S-, and N-genes	LAMP	22 min	30 min	50 copies/reaction	LFB, fluorescence	Streptavidin	Anti-conjugate antibody	Purchased from Milenia Biotech.	USD < 3	[30]
ENHANCE	N gene	LAMP	20–30 min	40–60 min	3 copies/μL	LFB, fluorescence	Avidin	Anti-conjugate antibody	Purchased from Milenia Biotech.	USD < 3	[29]
TESTOR	ORF1ab and N gene	RPA	-	120 min	5 copies/reaction	LFB, fluorescence	Anti-digoxin antibody	Anti-FAM antibody	Designed by Gong et al. and assembled by Twist Dx Ltd.	USD < 3	[31]
COVID-19 MCCD	ORF1ab and NP genes	MCDA	35 min	60 min	7 copies/μL	LFB, fluorescence	Anti-FITC antibody	Biotin-bovine serum albumin	Designed and assembled by Zhu et al.	-	[25]
iSCAN	N and E genes	LAMP	20 min	40 min	10 copies/reaction	LFB, fluorescence	Streptavidin	Anti-conjugate antibody	Purchased from Milenia Biotech.	USD < 3	[28]

## Data Availability

Data are contained within the article or Supplementary Materials.

## Supplementary Materials

The following are available online at https://www.mdpi.com/article/10.3390/bios12010011/s1, Figure S1: Test line signal optimization. (a) Assessment of different lengths of reporters with thymine and adenine base extension. (b) Assessment of different lengths of capture probes with adenine base extension, Figure S2: CRICOLAP RT-LAMP optimization (primer selection, temperature optimization, and reaction time optimization), Figure S3: Selection of crRNAs, Figure S4: CRICOLAP sensitivity evaluation. RT-LAMP sensitivity with or without loop primers, Figure S5: Validation of 60 clinical patient nasopharyngeal swabs using a commercial RT-PCR kit, Figure S6: LFA validation for S gene, Figure S7: LFA validation for N gene, Table S1: Selected oligonucleotide sequences (optimal) used in this study targeting S and N genes of COVID-19, Table S2: crRNAs used in this study, Table S3: LAMP primers used in this study, Table S4: List of reporter sequences used in this study, Table S5: List of capture probes used in this study, Table S6: Concordance comparison between CRICOLAP and RT-qPCR for 60 patients’ samples.
