# Supplementary material for: Rational Programming of Cas12a for Early-Stage Detection of COVID-19 by Lateral Flow Assay and Portable Real-Time Fluorescence Readout Facilities
Source: Biosensors (Basel). 2021 Dec 26;12(1):11. doi: 10.3390/bios12010011 (PMC8773725; doi:10.3390/bios12010011)
Supplement: Supplementary file 1 [file biosensors-12-00011-s001.zip › biosensors-1430393-supplementary.pdf]

biosensors

MDPI

Supplementary

Rational Programming of Cas12a for Early-Stage Detection of COVID-19 by Lateral Flow Assay and Portable Real-time Fluorescence Readout Facilities

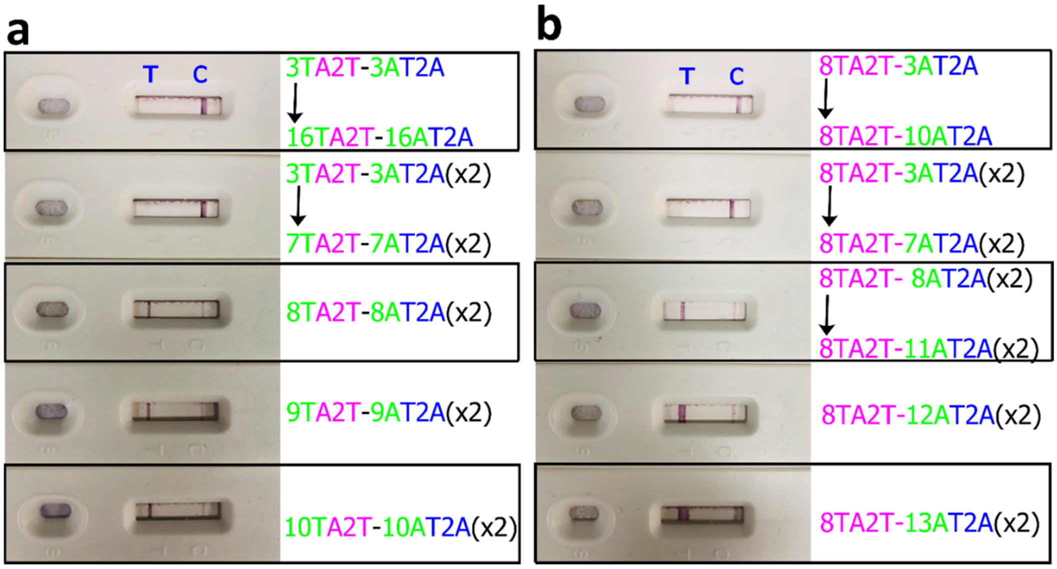

Figure S1. Test line signal optimization. (a) Assessment of different lengths of reporters with thymine and adenine bases extension. (b) Assessment of different lengths of capture probes with adenine base extension. Oligos in a purple color represent the reporter, and the ones in a blue color stand for the capture probe. The green color represents the variable section of thymine or adenine bases extension. (x2) symbolizes a doubled capture probe. Refer to table S4 for more details.

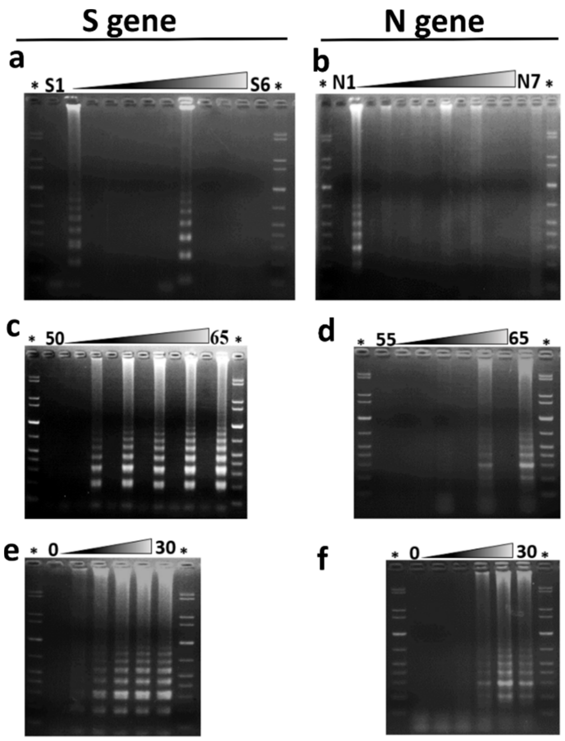

Figure S2. CRICOLAP RT-LAMP optimization. Gel electrophoresis of RT-LAMP products for S and N genes. The first and second columns represent S and N genes, respectively. S gene (a) and N gene (b) primer selection, two consecutive lanes

represent one primer set with negative and positive loadings. Temperature optimization (c, d), two consecutive lanes represent one temperature with the negative and positive samples from 50 to 65°C. Reaction time optimization (e, f). * stands for 1kb plus DNA marker.

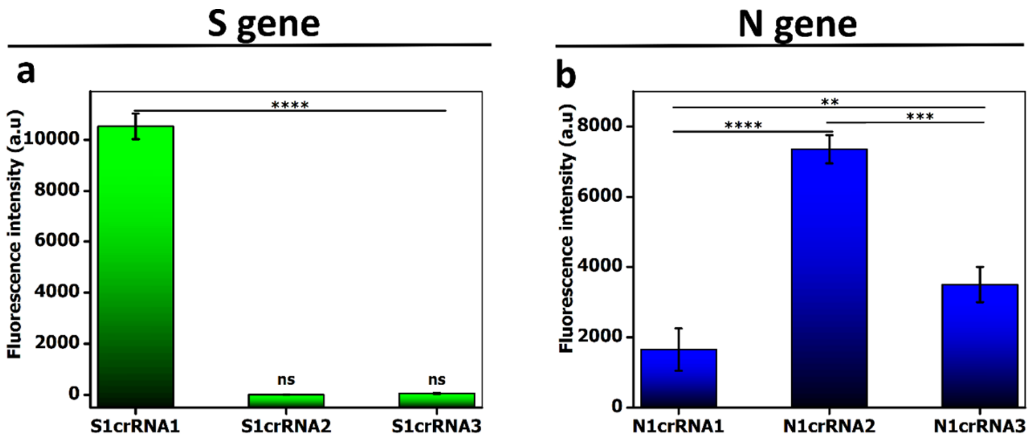

Figure S3. Selection of crRNAs (a, b). ns: non-significant different, $^{**}P < 0.01$, $^{***}P < 0.001$ $^{****}P < 0.0001$; two-tailed Student's *t*-test.

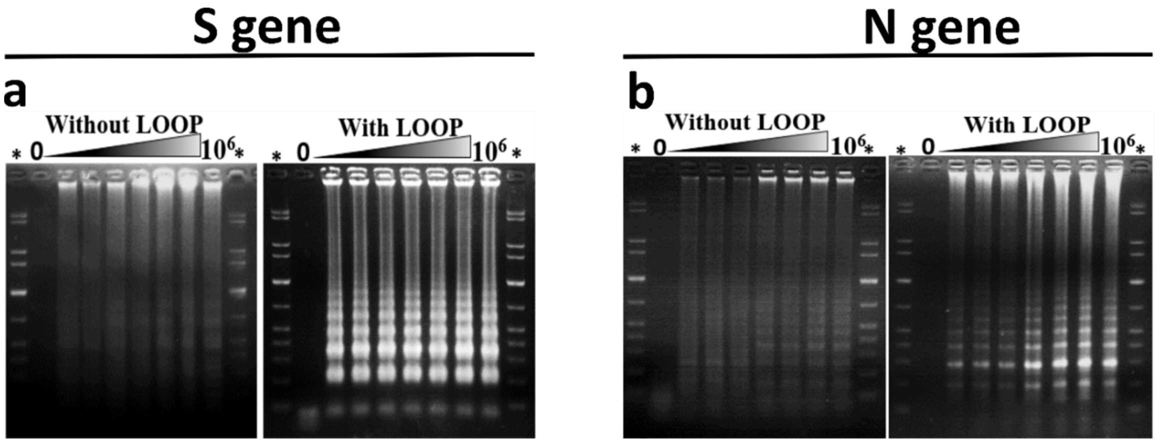

Figure S4. CRICOLAP sensitivity evaluation. RT-LAMP sensitivity with or without Loop primers (a, b). Each reaction was performed using 2 µL of the following serial dilutions: 0, 1, 10, 10^2,10^3,10^4,10^5, and 10^6 copies/µL. (*) stands for 1kb plus marker.

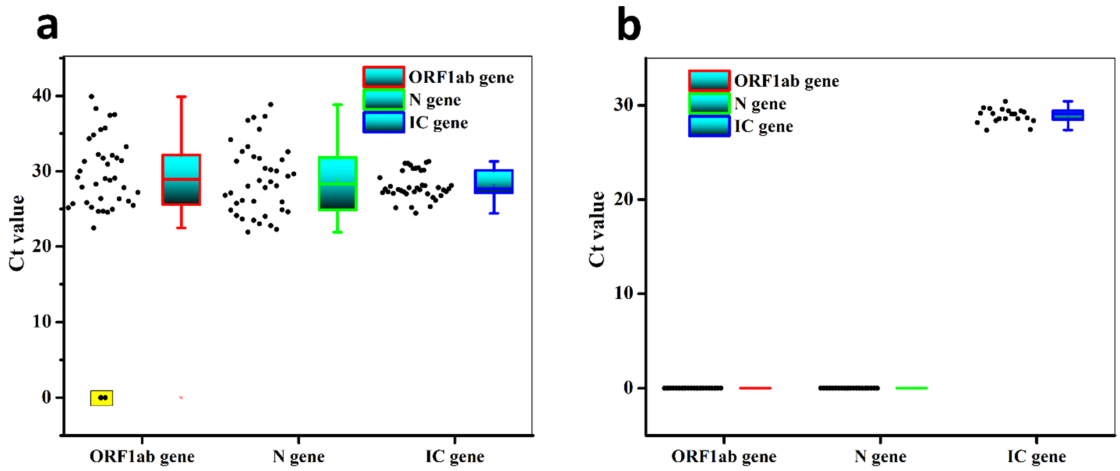

Figure S5. Validation of 60 clinical patient nasopharyngeal swabs using a Commercial RT-PCR kit. (a) Of the 40 positive samples examined, patients' samples number 38 and 39 (two outliers in a yellow box) were negative for the ORF1ab gene tested by a commercial RT-PCR kit; however, they were recognized positive for N and IC genes as well as S and N genes of our CRICOLAP (Fig. 5 A1, B1, A2, B2), Fig. S6 and Fig.S7. (b) 20 negative clinical samples. IC= internal control human gene.

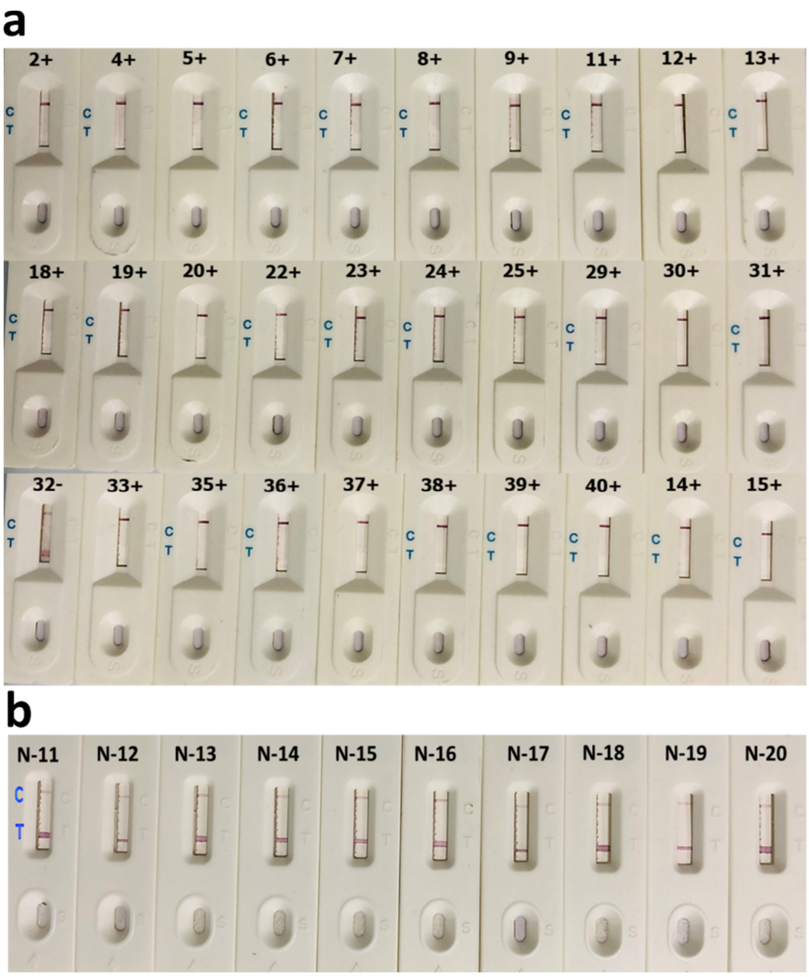

Figure S6. LFA validation for S-gene. (a) and (b) represent positive and negative patients' samples, respectively. Compared with a commercial RT-PCT kit, the LFA for S gene was 97.5% (39 out of 40 samples analyzed) consistent due to the sample number 32. Samples denoted with "+" and "N-" are from positive and negative clinical samples, respectively.

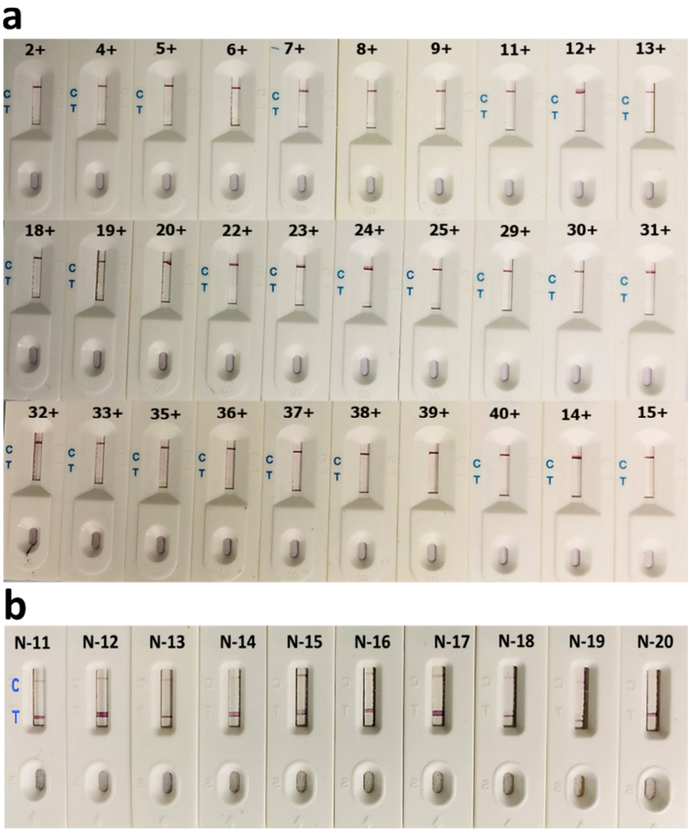

Figure S7. LFA validation for N-gene. (a) and (b) represent positive and negative patients' samples, respectively. Compared with a commercial RT-PCT kit, the LFA for N gene was 100% consistent in 60 samples analyzed. Samples denoted with "+" and "N-" are from positive and negative clinical samples, respectively.

Table S1. Selected oligonucleotide sequences (optimal) used in this study targeting S and N genes of the COVID-19.

Oligonucleotide	Sequence (5'-3')	Reference
Target genes	S gene<br>CACCACCAATTAAAGATTTTGGTGGTTTTAATTTTTCACAAATATTAC<br>CAGATCCATCAAAACCAAGCAAGAGGTCATTTATTGAAGATCTACT<br>TTTCAACAAAGTGACACTTGCAGATGCTGGCTTCATCAAACAATATG<br>GTGATTGCCTTGGTGATATTGCTGCTAGAGACCTCATTTGTGCACAA<br>AAGTTTAACGGCCTTACTGTTTTGCCACCTTTGCTCACAGATGAAAT<br>GATTGCTCAATACACTTCTGCACTGTTAGCGGGTACAATCACTTCTG<br>GTTGGACCTTTGGTGCAGGTGCTGCATTACAAATACCATTTGCTATG<br>CAAATGGCTTATAGGTTTAATGGTATTGGAGTTACACAGAATGTTCT<br>CTATG<br>N gene<br>GGTTGCAACTGAGGGAGCCTTGAATACACCAAAAGATCACATTGGC<br>ACCCGCAATCCTGCTAACAATGCTGCAATCGTGCTACAACTTCCTCA<br>AGGAACAACATTGCCAAAAGGCTTCTACGCAGAAGGGAGCAGAGG<br>CGGCAGTCAAGCCTCTTCTCGTTCCTCATCACGTAGTCGCAACAGTT<br>CAAGAAATTCAACTCCAGGCAGCAGTAGGGGAACTTCTCCTGCTAG<br>AATGGCTGGCAATGGCGGTGATGCTGCTCTTGCTTTGCTGCTGCTTG<br>ACAGATTGAACCAGCTTGAGAGCAAAATGTCTGGTAAAGGCCAACA<br>ACAACAAGGCCAAACTGTCACTAAGAAATCTGCTGCTGAGGC	This study
Expected amplicons	S-gene amplicon (258 bp)<br>AAACCAAGCAAGAGGTCATTTATTGAAGATCTACTTTTCAACAAAG<br>TGACACTTGCAGATGCTGGCTTCATCAAACAATATGGTGATTGCCTT<br>GGTGATATTGCTGCTAGAGACCTCATTTGTGCACAAAAGTTTAACGG	This study

	CCTTACTGTTTTGCCACCTTTGCTCACAGATGAAATGATTGCTCAATA<br>CACTTCTGCACTGTTAGCGGGTACAATCACTTCTGGTTGGACCTTTGG<br>TGCAGGTGCTGCATTACAAATA<br>N-gene amplicon (245 bp)<br>TCCTGCTAACAATGCTGCAATCGTGC-<br>TACAACTTCCTCAAGGAACAACATTGCCAAAAGGCTTCTAC-<br>GCAGAAGGGAGCAGAGGCGGCAGTCAA-<br>GCCTCTTCTCGTTCCTCATCACGTAGTCGCAACAGTTCAA-<br>GAAATTCAACTCCAGGCAGCAGTAGGGGAACTTCTCCTGCTA-<br>GAATGGCTGGCAATGGCGGTGATGCTGCTCTTGCTTTGCTGCTGCTT-<br>GACAGATTGAACCAGCTTGAGA	
Sequence (S+N genes) cloned in pcDNA 3.1+	AAGCTTGGTTGCAACTGAGGGAGCCTTGAATACACCAAAAGATCAC<br>ATTGGCACCCGCAATCCTGCTAACAATGCTGCAATCGTGCTACAACT<br>TCCTCAAGGAACAACATTGCCAAAAGGCTTCTACGCAGAAGGGAGC<br>AGAGGCGGCAGTCAAGCCTCTTCTCGTTCCTCATCACGTAGTCGCAA<br>CAGTTCAAGAAATTCAACTCCAGGCAGCAGTAGGGGAACTTCTCCT<br>GCTAGAATGGCTGGCAATGGCGGTGATGCTGCTCTTGCTTTGCTGCT<br>GCTTGACAGATTGAACCAGCTTGAGAGCAAAATGTCTGGTAAAGGC<br>CAACAACAACAAGGCCAAACTGTCACTAAGAAATCTGCTGCTGAGG<br>CGAATTCCACCACCAATTAAAGATTTTGGTGGTTTAATTTTTCACAA<br>ATATTACCAGATCCATCAAAACCAAGCAAGAGGTCATTTATTGAAG<br>ATCTACTTTTCAACAAAGTGACACTTGCAGATGCTGGCTTCATCAAA<br>CAATATGGTGATTGCCTTGGTGATATTGCTGCTAGAGACCTCATTTGT<br>GCACAAAAGTTTAACGGCCTTACTGTTTTGCCACCTTTGCTCACAGA<br>TGAAATGATTGCTCAATACACTTCTGCACTGTTAGCGGGTACAATCA<br>CTTCTGGTTGGACCTTTGGTGCAGGTGCTGCATTACAAATACCATTTG<br>CTATGCAAATGGCTTATAGGTTTAATGGTATTGGAGTTACACAGAAT<br>GTTCTCTATGCTCGAG	This study
F3	TTGGTGCAGGTATATGCG/ TCCTGCTAACAATGCTGC	This study
B3	ACATTGTACAATCTACTGATGTC/ TCTCAAGCTGGTTCAATCTG	This study
FIP	TAGGCAATGATGGATTGACTAGCTATTATCAGACTCAGACTAATTCT<br>CC/ AACGAGAAGAGGCTTGACTGCTCAAGGAACAACATTGCCA	This study
BIP	AACTCTATTGCCATACCCACAAATTTGGTCATAGACACTGGTAG/<br>CTCATCACGTAGTCGCAACAGTATTGCCAGCCATTCTAGC	This study
LoopF	GATTGACTAGCTACACTACGTG/ CCTTCTGCGTAGAAGCCTT	This study
LoopB	GCAGAAAATTCAGTTGCTTACT/ AATTCAACTCCAGGCAGCA	This study
Forward primer	TTGGTGCAGGTATATGCG/ TCCTGCTAACAATGCTGC	This study
Reverse primer	ACATTGTACAATCTACTGATGTC/ TCTCAAGCTGGTTCAATCTG	This study
crRNA	UAAUUUCUACUAAGUGUAGAUGUGCACAAAUGAGGUCUCUAGCA<br>G/UAAUUUCUACUAAGUGUAGAUGGCAAUGUUGUUCCUUGAGGA<br>AGU	This study
Activator	ATTTGTGCACAAAAGTTTAACGGCC/<br>CAACATTGCCAAAAGGCTTCTACGC	This study
ssDNA Reporters	Bio-TTTTTTTTATT, FAM-TTTTTTTTATT-TAMRA	This study
Test line capture probe	AATAAAAAAAAAAAAAAATAAAAAAAAAAAAA	This study

The N gene was inserted into the pcDNA3.1+ vector by HindIII/EcoRI double digestion, and the S gene was inserted behind the N gene through the EcoRI/XhoI cleavage site (bold).

Bio stands for 5' biotinylation, FAM and TAMRA are fluorescent and quencher labels at 5'-3' ends, respectively.

Underline represents crRNA targeting sequence.

The underlined sequence represents a capture probe sequence, 13A-T-2A (repeated twice).

Slash separates S gene and N gene corresponding oligo, respectively.

Table S2. crRNAs used in this study.

Gene	crRNAs	Sequence (5'-3')	Reference
S	crRNA1/NTS1	UAAUUUCUACUAAGUGUAGAUGUGCACAAAUGAGGUCUCUAG-CAG/ ATTTGTGCACAAAAGTTTAACGGCC	This study
	crRNA2/NTS2	UAAUUUCUACUAAGUGUAGAUACGGCCUUACUGUUUUGCCACCUU/ TGCACAAAAGTTTAACGGCCTTACT	This study
	crRNA3/NTS3	UAAUUUCUACUAAGUGUAGAUGCCACCUUUGCUCACAGAUGAAAU /GCACAAAAGTTTAACGGCCTTACTG	This study
N	crRNA1/NTS1	UAAUUUCUACUAAGUGUAGAUGCAAUGUUGUUCCUUGAGGAAGU U/ ACAACATTGCCAAAAGGCTTCTACG	This study
	crRNA2/NTS2	UAAUUUCUACUAAGUGUAGAUGGCAAUGUUGUUCCUUGAGGAAG U/ CAACATTGCCAAAAGGCTTCTACGC	This study
	crRNA3/NTS3	UAAUUUCUACUAAGUGUAGAUUUGAACUGUUGCGACUACGUGAU G/ AACAGTTCAAGAAATTCAACTCCAG	This study

NTS stands for non-target strand (activator)

Table S3. RT-LAMP primers used in this study.

Primers	Sequence (5'-3')	Referen
S1F3	AAACCAAGCAAGAGGTCATT	This study
S1B3	GTATTTGTAATGCAGCACCTG	
S1FIP	TGCACAAATGAGGTCTCTAGCACAGATGCTGGCTTCATCAA	
S1BIP	TTGCCACCTTTGCTCACAGATTCCAACCAGAAGTGATTGTAC	
S1LoopF	CAATATCACCAAGGCAATCACC	
S1LoopB	TGATTGCTCAATACACTTCTGC	
S2F3	TGCTGCTAGAGACCTCAT	This study
S2B3	CAATACCATTAAACCTATAAGCCAT	
S2FIP	TTGAGCAATCATTTCATCTGTGAGCTTGTGCACAAAAGTTTAACGG	
S2BIP	GCACTGTTAGCGGGTACAATCATAGCAAATGGTATTTGTAATGC	
S3F3	TTCATCAAACAATATGGTGATTG	This study
S3B3	AGCAAATGGTATTTGTAATGCA	
S3FIP	GTGGCAAAACAGTAAGGCCGCCTTGGTGATATTGCTGCTA	
S3BIP	CTTTGCTCACAGATGAAATGATTGCCCAAAGGTCCAACCAGAA	
S4F3	TTGGTGCAGGTATATGCG	This study
S4B3	ACATTGTACAATCTACTGATGTC	
S4FIP	TAGGCAATGATGGATTGACTAGCTATTATCAGACTCAGACTAATTCTCC	
S4BIP	AACTCTATTGCCATACCCACAAATTTGGTCATAGACACTGGTAG	
S4LoopF	GATTGACTAGCTACACTACGTG	
S4LoopB	GCAGAAAATTCAGTTGCTTACT	
S5F3	CTCTATTGCCATACCCACA	This study
S5B3	CTTGTGCAAAAACTTCTTGG	
S5FIP	CATTCAGTTGAATCACCACAAATGTGTGTTACCACAGAAATTCTACC	
S5BIP	GTTGCAATATGGCAGTTTTTGTACATTTTGTCTTGTTCAACAGCT	
S6F3	AAACAATTTGGCAGAGACATTG	This study
S6B3	CAAGTAGGAGTAAGTTGATCTG	
S6FIP	GACACCACCAAAAGAACATGGTGCTGACACTACTGATGCTGTC	
S6BIP	CCAGGAACAAATACTTCTAACCAGGTGAATAGCAACAGGGACTT	
N1F3	TCCTGCTAACAATGCTGC	This study
N1B3	TCTCAAGCTGGTTCAATCTG	
N1FIP	AACGAGAAGAGGCTTGACTGCTCAAGGAACAACATTGCCA	

N1BIP	CTCATCACGTAGTCGCAACAGTATTGCCAGCCATTCTAGC	
N1LoopF	CCTTCTGCGTAGAAGCCTT	
N1LoopB	AATTCAACTCCAGGCAGCA	
N2F3	TGGCTACTACCGAAGAGCT	This study
N2B3	TGCAGCATTGTTAGCAGGAT	
N2FIP	TCTGGCCCAGTTCCTAGGTAGTGACGAATTCGTGGTGGTGA	
N2BIP	AGACGGCATCATATGGGTTGCA-GCGGGTGCCAATGTGATC	
N3F3	AGATCACATTGGCACCCG	This study
N3B3	CCATTGCCAGCCATTCTAGC	
N3FIP	TGCTCCCTTCTGCGTAGAAGCCAATGCTGCAATCGTGCTAC	
N3BIP	GGCGGCAGTCAAGCCTCTTCCCTACTGCTGCCTGGAGTT	
N4F3	GCCAAAAGGCTTCTACGCA	This study
N4B3	TTGCTCTCAAGCTGGTTCAA	
N4FIP	TCCCCTACTGCTGCCTGGAGGCAGTCAAGCCTCTTCTCG	
N4BIP	TCTCCTGCTAGAATGGCTGGCATCTGTCAAGCAGCAGCAAAG	
N5F3	TGGACCCCAAAATCAGCG	This study
N5B3	GCCTTGTCCTCGAGGGAAT	
N5FIP	CCACTGCGTTCTCCATTCTGGTAAATGCACCCCGCATTACG	
N5BIP	CGCGATCAAAACAACGTCGGCCCTTGCCATGTTGAGTGAGA	
N6F3	CCAGAATGGAGAACGCAGTG	This study
N6B3	CCGTCACCACCACGAATT	
N6FIP	AGCGGTGAACCAAGACGCAGGGCGCGATCAAAACAACG	
N6BIP	AATTCCCTCGAGGACAAGGCGAGCTCTTCGGTAGTAGCCAA	
N7F3	AACACAAGCTTTCGGCAG	This study
N7B3	GAAATTTGGATCTTTGTCATCC	
N7FIP	TGCGGCCAATGTTTGTAATCAGCCAAGGAAATTTTGGGGAC	
N7BIP	CGCATTGGCATGGAAGTCACTTTGATGGCACCTGTGTAG	
N7LoopF	TTCCTTGTCTGATTAGTTC	
N7LoopB	ACCTTCGGGAACGTGGTT	

Table S4. The list of reporter sequences used in our study.

Name	Reporter sequence (5′-3′)
Bio-3T-A-2T	Biotin-TTTATT
Bio-4T-A-2T	Biotin-TTTTATT
Bio-5T-A-2T	Biotin-TTTTTATT
Bio-6T-A-2T	Biotin-TTTTTTATT
Bio-7T-A-2T	Biotin-TTTTTTTATT
Bio-8T-A-2T*	Biotin-TTTTTTTTATT
Bio-9T-A-2T	Biotin-TTTTTTTTTATT
Bio-10T-A-2T	Biotin-TTTTTTTTTTATT
Bio-11T-A-2T	Biotin-TTTTTTTTTTTATT
Bio-12T-A-2T	Biotin-TTTTTTTTTTTTATT
Bio-13T-A-2T	Biotin-TTTTTTTTTTTTTATT
Bio-14T-A-2T	Biotin-TTTTTTTTTTTTTTATT
Bio-15T-A-2T	Biotin-TTTTTTTTTTTTTTTATT
Bio-16T-A-2T	Biotin-TTTTTTTTTTTTTTTTATT

Underlined sections represent the optimized variable bases of poly-thymine for the reporter optimization. (*) represents the optimum reporter.

Table S5. The list of capture probe sequences used in our study.

Name	Capture probe sequence (3′-5′)
3A-T-2A	AAATAA
4A-T-2A	AAAATAA
5A-T-2A	AAAAATAA
6A-T-2A	AAAAAATAA
7A-T-2A	AAAAAAATAA
8A-T-2A	AAAAAAAATAA
9A-T-2A	AAAAAAAAATAA
10A-T-2A	AAAAAAAAAATAA
11A-T-2A	AAAAAAAAAAATAA
12A-T-2A	AAAAAAAAAAAATAA
13A-T-2A	AAAAAAAAAAAAATAA
14A-T-2A	AAAAAAAAAAAAAATAA
15A-T-2A	AAAAAAAAAAAAAAATAA
16A-T-2A	AAAAAAAAAAAAAAAATAA
3A-T-2A (x2)	AAATAAAAATAA
4A-T-2A (x2)	AAAATAAAAAATAA
5A-T-2A (x2)	AAAAATAAAAAAATAA
6A-T-2A (x2)	AAAAAATAAAAAAAATAA
7A-T-2A (x2)	AAAAAAATAAAAAAAAATAA
8A-T-2A (x2)	AAAAAAAATAAAAAAAAAATAA
9A-T-2A (x2)	AAAAAAAAATAAAAAAAAAAATAA
10A-T-2A (x2)	AAAAAAAAAATAAAAAAAAAAAATAA
11A-T-2A (x2)	AAAAAAAAAAATAAAAAAAAAAAAATAA
12A-T-2A (x2)	AAAAAAAAAAAATAAAAAAAAAAAAAATAA
13A-T-2A (x2)**	AAAAAAAAAAAAATAAAAAAAAAAAAAAATAA

Underlined sections represent the optimized variable bases of poly-adenine for the capture probe optimization. (**) represents the optimum capture probe. (x2) symbolizes a doubled capture probe.

Table S6. Concordance comparison between CRICOLAP and RT-qPCR for 60 patients' samples.

	RT-PCR validated positive patients' samples		RT-PCR validated negative patients' samples	
TOTAL NUMBER OF SAMPLES IN THIS STUDY	40		20	
SARS-CoV-2 gene targets	S gene	N gene	S gene	N gene
CRICOLAP (real-time fluorescence detection)	39	40	20	20
CRICOLAP (LFS detection)	39	40	20	20
Concordance (real-time fluorescence detection)	97.5%	100%	100%	100%
Concordance (LFS detection)	97.5%	100%	100%	100%
